# Observed Prevalence and Characterization of Fluoroquinolone-Resistant and Multidrug-Resistant Bacteria in Loggerhead Sea Turtles (*Caretta caretta*) from the Adriatic Sea

**DOI:** 10.3390/antibiotics14030252

**Published:** 2025-03-01

**Authors:** Olimpia Lai, Antonella Tinelli, Simona Soloperto, Giuseppe Crescenzo, Domenico Galante, Angela Calarco, Magda Tribuzio, Viviana Manzulli, Giulia Caioni, Claudia Zizzadoro, Antonella Damiano, Antonio Camarda, Nicola Pugliese

**Affiliations:** 1 Dipartimento di Medicina Veterinaria, Università degli Studi di Bari, 70010 Valenzano, Italy; olimpia.lai@uniba.it (O.L.); antonella.tinelli@uniba.it (A.T.); giuseppe.crescenzo@uniba.it (G.C.); a.calarco@studenti.uniba.it (A.C.); magda.tribuzio@outlook.it (M.T.); claudia.zizzadoro@uniba.it (C.Z.); antonio.camarda@uniba.it (A.C.); 2 Centro Recupero Tartarughe Marine “Luigi Cantoro”, Torre Guaceto, 72012 Carovigno, Italy; solopertosimona@gmail.com; 3 Istituto Zooprofilattico Sperimentale della Puglia e della Basilicata, 71121 Foggia, Italy; domenico.galante@izspb.it (D.G.); viviana.manzulli@izspb.it (V.M.); 4 Department of Bioscience and Technology for Food, Agriculture and Environment, University of Teramo, 64100 Teramo, Italy; gcaioni@unite.it (G.C.); adamiano@unite.it (A.D.)

**Keywords:** *Caretta caretta*, antimicrobial resistance, marine ecosystem, environment, fluoroquinolones, *Vagococcus*, multidrug resistance

## Abstract

**Background/Objectives**: Antimicrobial resistance (AMR) is a major global health concern with profound implications for human, animal, and environmental health. Marine ecosystems are emerging as reservoirs of resistant bacteria due to contamination from anthropogenic activities. This study aimed to investigate fluoroquinolone-resistant and multidrug-resistant bacteria in loggerhead sea turtles (*Caretta caretta*). **Methods**: Cloacal swabs were collected from 28 loggerhead sea turtles at a rescue center in southern Italy. Swabs were cultured in nutrient media supplemented with enrofloxacin. Bacterial isolates underwent identification by MALDI-TOF, antimicrobial susceptibility testing, and assessment for multidrug resistance. Conjugation experiments evaluated the transferability of enrofloxacin resistance. **Results**: Thirty-six enrofloxacin-resistant bacterial strains were isolated from 22 turtles. The identified species included *Vagococcus fluvialis* (13 strains), *Citrobacter freundii* (5), *Escherichia coli* (6), and *Pseudomonas mendocina* (4). Thirty-five isolates exhibited multidrug resistance, with resistance to critically important antibiotics such as imipenem observed in *C. freundii* and *Enterobacter faecium*. Conjugation experiments showed no transfer of resistance genes. **Conclusions**: The study highlights the prevalence of fluoroquinolone-resistant and multidrug-resistant bacteria in *C. caretta*, implicating marine environments as reservoirs of AMR. The findings underscore the need for stricter regulation of antimicrobial use and monitoring of resistance dissemination in marine ecosystems. These results contribute to understanding AMR dynamics within the One Health framework, emphasizing the interconnectedness of environmental, animal, and human health.

## 1. Introduction

Antimicrobial resistance (AMR) has been identified by the World Health Organization (WHO) as a critical threat to global health, food security, and development [1]. It is estimated that AMR could cause 10 million deaths annually by 2050 and push an additional 24 million people into extreme poverty over the next decade if significant interventions are not implemented [2].

Infections caused by resistant bacteria are associated with increased morbidity, mortality, and healthcare costs compared to infections caused by susceptible strains [3]. This is true for both humans and animals, as patients with resistant infections often require more extended hospital stays, more intensive care, and treatment with more toxic, expensive, or inaccessible drugs [4].

In the One Health framework, which emphasizes the interconnectedness of human, animal, and environmental health, resistant bacteria can be transmitted across species and ecosystems [5]. Human pharmaceutical residues are usually detected in environmental waters [6], and antimicrobials have often been identified [7]. Several routes of AMR diffusion are known, including livestock, where the use of antibiotics can lead to the emergence of resistant strains that can be, in turn, transmitted to humans through direct contact or the consumption of contaminated food products [8]. Similarly, the use of antibiotics in aquaculture can contribute to the spread of resistance in marine environments, with potential repercussions for both human and animal health [9].

Furthermore, environmental contamination with antimicrobials and resistant bacteria via agricultural runoff, sewage, and waste can introduce, select, or promote the proliferation of resistant strains in natural ecosystems, which can subsequently reach humans and animals through different pathways, including water and soil [10].

The role of terrestrial and marine ecosystems as reservoirs for resistant bacteria is an area of growing concern. Interaction between wildlife, domestic animals, and humans can facilitate the transmission of resistant bacteria across species and ecosystems, especially in overlapping habitats [11].

Marine environments are particularly significant in this context, as aquatic ecosystems are exposed to antimicrobial residues from human activities, together with a wide pool of resistance genes selected in humans or domestic animals, thus fostering the development and spread of resistance among aquatic bacteria [12].

Fluoroquinolones are among the antimicrobials frequently detected in contaminated aquatic environments [13]. That class of broad-spectrum antibacterials is considered critically important in human medicine [14], and such antibacterials have been classified by the European Medicines Agency in category B, which encompasses antimicrobials that should only be used when alternatives in lower categories are clinically ineffective [15]. This classification of fluoroquinolones reflects concerns over the emergence and spread of resistant bacterial strains, which pose significant challenges to public health [16,17,18].

Fluoroquinolone resistance is primarily mediated by multiple mutations in bacterial DNA gyrase and topoisomerase IV, encoded by *gyrA*, *gyrB*, *parC*, and *parE* genes, respectively [19]. Plasmid-mediated resistance genes have been increasingly detected and reported in the last decade, raising further concern in the scientific community and regulatory authorities [20].

Considering the critical importance of fluoroquinolones and their wide use in both human and veterinary medicine, and recognizing the concern for the even wider diffusion of resistant bacterial strains, this study aimed to investigate the presence and distribution of fluoroquinolone-resistant bacteria in the marine environment, where the selective pressure due to the direct exposure of bacteria to antimicrobials should not be relevant. Considering the dilution effect of seawater, the study focused on bacteria isolated from the cloaca of loggerhead sea turtles (*Caretta caretta*), which live and feed in marine environments and can thus concentrate bacteria of interest in their gastrointestinal tracts.

## 2. Results

A total of 36 enrofloxacin (ENR)-resistant strains were isolated from 22 out of the 28 loggerhead sea turtles sampled. Among those, 13 were identified as *Vagococcus* (*Va.*) *fluvialis*, 6 as *Escherichia coli*, 5 as *Citrobacter freundii*, and 4 as *Pseudomonas mendocina*. Additionally, there was one strain each of *Aeromonas caviae*, *Aeromonas veronii*, *Enterococcus faecium*, *Morganella morganii*, *Staphylococcus haemolyticus*, and *Vibrio* (*Vi.*) *fluvialis*. One isolate, identified by MALDI-TOF as *Klebsiella pneumoniae*, was identified as *Klebsiella variicola* subsp. *variicola* (hereafter, *K. variicola*), following an analysis of the 16S rRNA nucleotide sequence. Similarly, insolate FVBA8, initially identified by MALDI-TOF as *C. freundii*, was finally identified as *Citrobacter portucalensis* after the 16S rRNA gene analysis. The identification of the other five *C. freundii* strains was confirmed by the 16S rRNA gene. The MIC values for ENR ranged from 16 to 256 μg/mL (Table 1), with the highest resistance being observed in *E. coli*, *P. mendocina*, and *M. morganii* strains.

The resistance to ENR was found to be not transferable to the recipient strains in any of the conjugation experiments.

Multidrug resistance was identified in 35 strains. Specifically, *K. variicola* and several strains of *Va. fluvialis* exhibited resistance to seven–nine antibiotics. Among the gut commensal of terrestrial animals, all the 5 *C. freundii* strains displayed resistance to five to seven antibiotics, with one being resistant to IPM, a critically important antibiotic.

No isolate was found to be resistant to CST, apart from FVBA19, identified as *M. morganii*, a species known to be intrinsically resistant to that antibiotic [21].

Resistance to the aminoglycoside STR was observed in 22 strains, while resistance to the other members of that class, namely AMK and GEN, was less common, as detected in four and five strains, respectively. All AMK-resistant strains were identified as *Va. fluvialis*, while GEN resistance was detected in three strains of *Va. fluvialis*, the *E. faecium* isolate, and the *K. variicola* strain.

**Figure 1:**
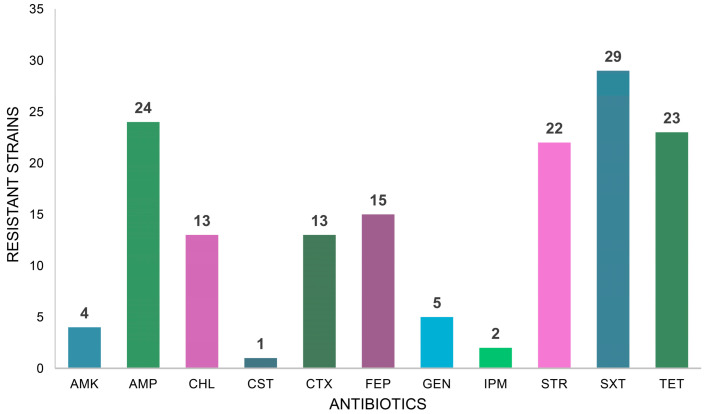
Distribution of resistances of the isolated strains to the tested antibiotics. AMK: amikacin, AMP: ampicillin, CHL: chloramphenicol, CST: colistin, CTX: cefotaxime, FEP: cefepime, GEN: gentamicin, IPM: imipenem, STR: streptomycin, SXT: sulfa-methoxazole/trimethoprim, TET: tetracycline.

Resistance to beta-lactams was also widely prevalent. Twenty-four strains were resistant to AMP, while 13 and 15 were resistant to the third and fourth-generation cephalosporins CTX and FEP, respectively (Figure 1). Finally, the majority of strains (29 out of 36) were resistant to SXT, and 23 were resistant to TET. Resistance to CHL, a phenicol, was identified in 13 strains (Figure 1).

## 3. Discussion

The study highlighted the prevalence of ENR-resistant bacterial strains in *C. caretta*. The identification of both Gram-positive (*Va. fluvialis*, *E. faecium*, *S. haemolyticus*) and Gram-negative species (*E. coli*, *C. freundii*, *C. portucalensis*, *P. mendocina*, *Vi. fluvialis*, *M. morganii*, *K. variicola*, and *A. veronii*) that are resistant to ENR and, in most cases, present an MDR profile demonstrates a wide circulation of resistant strains in the marine environment which are also relevant and of particular concern. Moreover, the cross-resistance frequently observed within the fluoroquinolone class [22] may further amplify the significance of these findings. Strains resistant to veterinary-only authorized compounds, such as enrofloxacin or danofloxacin, are usually resistant to other fluoroquinolones, including ciprofloxacin, levofloxacin, and norfloxacin [23,24]. Therefore, the ENR-resistant strains that can infect both humans and animals will likely be insensitive to fluoroquinolones used in human medicine.

The marine environment has increasingly been recognized as a reservoir for resistant bacterial strains and genetic determinants of drug resistance [25]. Anthropogenic activities, such as wastewater discharge, agricultural runoff, and aquaculture practices, introduce antibiotics, resistant bacteria, and resistance genes into coastal waters, thus exerting a selective pressure that favors the survival and proliferation of MDR strains [12].

The isolation of species commonly associated with the guts of terrestrial and aquatic animals, such as *E. coli*, *C. freundii*, and *M. morganii*, alongside species typical of aquatic ecosystems, like *P. mendocina* and *A. veronii*, suggests that resistant strains from different ecological niches may converge in the marine environment. Additionally, the circulation of opportunistic pathogens, such as *Vi. fluvialis* and *Va. fluvialis*, may impact public health, as these species are being increasingly reported as emerging pathogens [26,27]. Humans can come into contact with resistant strains dispersed in the marine environment mostly through recreational water activities, seafood consumption, occupational exposure, or proximity to animal reservoirs [28]. While the transfer of resistant strains from humans and farmed animals to the environment is thoroughly documented, the extent of the reverse process is less well established [28]. Nonetheless, an increasing amount of available data supports this trend; for example, extended-spectrum β-lactamase-producing *E. coli* or *K. pneumonia* strains have been more frequently isolated in swimmers [29], and the dissemination of *Enterococcus faecalis* strains resistant to critically important antimicrobials has also been associated with their environmental circulation [30].

The detection of an MDR *E. faecium* strain resistant to IPM in this study aligns with the species’ well-documented role in nosocomial infections [31]. The observed broad resistance spectrum is characteristic of *Enterococcus* spp., whose resistance to several critically important antibiotics, including antimicrobial peptides and fluoroquinolones, is increasingly recognized [32]. Intrinsic resistance to cephalosporins has also been reported [33]. Despite belonging to the *Enterococcaceae* family, no specific literature exists for the *Vagococcus* genus at the time of writing. In this study, some *Va. fluvialis* strains were found that were resistant to FEP (fourth-generation cephalosporin) but not to CTX (third-generation cephalosporin), and all the strains were susceptible to AMP. This pattern strongly suggests that intrinsic resistance to certain cephalosporins may also be a distinctive feature of *Va. fluvialis*. Recently, this species has drawn attention due to its MDR patterns, which can include forms of fluoroquinolone resistance [34,35]. Interestingly, its genome harbors mobile or mobilizable genetic elements, such as plasmids, prophages, and insertion sequences [36]. However, to our knowledge, the association between these potentially transferable genetic elements and resistance genes remains poorly understood. Heterologous transmission of fluoroquinolone resistance to *S. aureus*, one of the most medically relevant species of the class *Bacilli*, has not been observed in this study, but ongoing experiments are exploring this possibility. On the other hand, multi-drug resistance was a typical hallmark of the *Va. fluvialis* samples isolated in this study, since most of them exhibited resistance to six or more antimicrobials, including SXT, TET, AMK, or GEN. Resistance to third- and fourth-generation cephalosporins (CTX and FEP) was also common, but no carbapenem resistance was detected.

Similarly, the isolation of a fluoroquinolone and cephalosporin-resistant *K. variicola* strain from *C. caretta* is noteworthy. Despite this species being typically associated with plants or fungi [37,38], it has recently been implicated in human cases, especially in nosocomial and community-acquired infections [39,40].

The significant increase in hospital infections caused by MDR strains, often originating from not strictly pathogenic species, is concerning both the scientific and political communities worldwide [41]. The American Centers for Disease Control and Prevention estimated that more than 35,000 deaths per year are attributable to MDR infections, a number which is expected to rise [2]. Notably, many of these infections are caused by opportunistic pathogens that exploit compromised immune systems [42]. Their resistance to multiple antibiotics, including last-resort treatments, complicates clinical management and results in increased morbidity, mortality, and healthcare costs [43].

In that framework, the detection of numerous MDR strains characterized by ENR resistance in sea turtles raises critical questions regarding the environmental reservoir of resistance. Since loggerhead sea turtles primarily inhabit open marine environments, direct antibiotic exposure is unlikely, while resistant strains may be disseminated through seawater. It remains unclear where and how those strains were selected or whether they have never undergone specific selective pressures. It is possible to speculate that strains of commensal species, such as *E. coli* or *C. freundii*, which exhibited the highest MIC for ENR and resistance to critically important antibiotics like ENR and IPM, may have originated from human or veterinary settings.

Conversely, the isolation of *P. mendocina*, *Vi. fluvialis*, *Va. fluvialis*, and *K. variicola* suggests that resistant strains may circulate and persist in the marine ecosystem independently of direct selective pressures. Those findings also underscore the widespread environmental dissemination of fluoroquinolone-resistant strains, even though such resistance typically requires multiple mutations in the *par* and *gyr* genes.

In light of these findings, the importance of strict and systematic monitoring should be stressed. Current controls of the microbiological quality of wastewater, agricultural, or manure runoffs primarily focus on ensuring the absence of pathogenic bacteria, but the systematic assessment of antimicrobial resistance has not yet been implemented [10]. Although often neglected, wastewater can serve not only as a hotspot for the diffusion of MDR bacteria but also as a melting pot for resistance gene transfering [44]. As evidenced by this study, dedicated monitoring is advisable because antimicrobial resistance is a feature shared by pathogenic, commensal, or environmental bacterial strains. The most recent genetic and genomic strategies and techniques may help evaluate the presence of antimicrobial resistance directly from water, and they can be applied to anthropogenic water returns or to environmental water collected for human use [45,46].

Therefore, in addition to global regulatory actions aimed at restricting the use of critically important antimicrobials and emphasizing the responsibility of medical and veterinary stakeholders to reserve those drugs for strictly necessary cases, efforts should also be dedicated to identifying, monitoring, and controlling the environmental sources of antimicrobial resistance. These sources may serve as reservoirs and points of emergence for MDR strains, including those resistant to critically important antimicrobials.

## 4. Materials and Methods

### 4.1. Bacterial Strains

The bacterial strains included in this study were isolated from cloacal swabs collected from 28 loggerhead sea turtles (*C. caretta*) admitted to the Sea Turtle Rescue Center in Torre Guaceto, southern Italy (latitude 40°43′20.2332″ N, longitude 17°46′22.7208″ E). The turtles were admitted due to traumatic lesions or entanglement in fishing nets; none presented infectious diseases. Usually, the animals were recovered by fishermen or yachters in the open sea, far from anthropized settlements, and brought to the rescue center within a short time. Cloacal swabs were collected by trained veterinarian staff as part of the routine screening upon admittance to the center. The procedure was not harmful to the animals, and none suffered injuries or any other lesions because of the sampling. Beached turtles were not included in the study because they were usually in poor health conditions, which could have imbalanced their cloacal bacterial population. Ethical approval for sample collection for subsequent studies was granted by the Ethics Committee of the Department of Veterinary Medicine at the University of Bari (authorization number 42/24).

The swabs were transported in a refrigerated container to the Department of Veterinary Medicine of the University of Bari and processed within 6 h of sampling. The swabs were included in a Luria–Bertani broth (LB) and incubated at 27 °C for 24 h. If no evident bacterial growth was observed, the broth was incubated for a further 24 h for a total incubation time of 48 h.

A loopful of each broth culture was streaked onto Luria–Bertani agar (LBA) plates supplemented with 10 μg/mL enrofloxacin (ENR, courtesy of Bayer Animal Health Italia, Milan, Italy) and incubated at 27 °C for 48 h. After incubation, colonies were inspected for their morphological features, and at least one representative colony of each morphotype was picked and re-streaked on LBA supplemented with ENR. After incubation, single, well-isolated colonies were used to establish pure cultures, which were stored in LB supplemented with 15% glycerol at −80 °C. Enrofloxacin-susceptible *Escherichia coli* ATCC 25,922 served as a quality control strain.

To obtain rifampicin (RIF)-resistant strains for conjugation experiments, overnight LB cultures of *E. coli* ATCC 25,922 and *Staphylococcus aureus* ATCC 13,565 were spread on LBA plates supplemented with 100 μg/mL RIF. Plates were incubated at 37 °C for 18 h, and single, well-isolated colonies were re-streaked twice on LBA supplemented with RIF to obtain pure cultures, which were stored in 15% glycerol at −80 °C.

### 4.2. Bacterial Identification

Wet mount microscopy and Gram staining were performed on mid-logarithmic phase broth cultures to assess morphology, motility, and Gram reaction.

Bacterial identification was carried out using matrix-assisted laser desorption/ionization–time of flight (MALDI-TOF) mass spectrometry. Briefly, fresh bacterial colonies, aerobically subcultured for 24 h at 37 °C in 5% sheep blood agar, were applied onto a 96-well steel target plate (Bruker Daltonics, Bremen, Germany) using a wooden toothpick. The spots were covered with 1 μL of 70% formic acid and allowed to dry at room temperature prior to the addition of 1 μL of matrix solution, α-cyano-4-hydroxycynnamic acid (HCCA, Bruker Daltonik GmbH, Bremen, Germany) prepared following the manufacturer’s instructions.

The mass spectra were acquired using a Microflex LT/SH™ mass spectrometer (Bruker Daltonik GmbH, Bremen, Germany), which was operated in a linear positive mode covering a mass-to-charge ratio (*m*/*z*) of between 2000 and 20,000 Da. Each spot of the target plate was hit with a pulsed nitrogen laser beam operating at 337 nm with a frequency equal to 60 Hz. After the laser shot, the gas phase ions obtained were accelerated in the flight tube by an acceleration voltage with optimized values for the mass range understudy.

Before the analysis, the instrument was calibrated in the broad molecular weight range between 2 and 20 kDa using the Bruker Bacterial Test Standard (BTS, Bruker Daltonik GmbH, Bremen, Germany). The data were processed automatically by MBT Compass 4.1.70 software (Bruker Daltonik GmbH, Bremen, Germany), and the mass spectra were compared with those of known microbial isolates of the commercial libraries provided by Bruker Daltonik. The results are expressed with log(score) values between 0 and 3.0, indicative of the matching between the sample spectrum and the MSPs in the reference database. A log(score) value < 1.7 indicates that it could not identify the bacteria strain; a log(score) value between 1.7 and 2.0 indicates that identification is reliable only at the genus level, while a log(score) value ≥2.0 indicates that identification is reliable at the species level of the microorganism.

Due to the limited resolution of MALDI-TOF for some species within the genus *Klebsiella*, the partial nucleotide sequence of the 16S rRNA gene was determined for seven strains (namely, FVBA8, FVBA22, FVBA23, FVBA24, FVBA25, FVBA26, and FVBA29) which were identified by MALDI-TOF as *Klebsiella pneumoniae*. Briefly, a single colony was lysed by heat in sterile distilled water and used as a template in a PCR carried out by using the universal primer pairs 27f (5′-AGAGTTTGATCMTGGCTCAG-3′)–1492r (5′-CTACGRVTACCTTGTTACGAC-3′) targeting the 16S rRNA gene [47]. Amplicons were purified by means of the PureLink PCR Purification Kit (ThermoFisher Scientific, Milan, Italy) and sequenced by the Big Dye Terminator (Applied Biosystems, Milan, Italy) at the facilities of BMR Genomics (Padua, Italy). Assembly was carried out by using the CAP3 Sequence Assembly Program [48] after trimming primers and low-quality regions. Taxonomic identification was achieved by using EzTaxon [49]. The sequences were submitted to GenBank under the accession numbers PV111306 (FVBA8), PV111307 (FVBA22), PV111308 (FVBA23), PV111309 (FVBA24), PV111310 (FVBA25), PV111311 (FVBA26), and PQ763953 (FVBA29).

### 4.3. Antimicrobial Susceptibility Test

The minimal inhibitory concentration (MIC) of ENR was determined by the agar dilution procedure for each strain following standard protocol for the dilution antimicrobial susceptibility tests [50]. Similarly, the MIC for CST has been determined for the Gram-negative strains.

The antimicrobial susceptibility profile of each isolate was assessed by the disk diffusion method in accordance with the Clinical and Laboratory Standards Institute guidelines. Strains were tested for their susceptibility to antibiotics commonly used in human and veterinary medicine, specifically amikacin (AMK), ampicillin (AMP), cefepime (FEP), cefotaxime (CTX), chloramphenicol (CHL), gentamicin (GEN), imipenem (IPM), streptomycin (STR), sulfamethoxazole/trimethoprim (SXT), and tetracycline (TET).

Strains which were resistant to three or more antibiotics, belonging to at least three or more classes, were classified as multidrug-resistant (MDR) [51].

### 4.4. Conjugation Experiments

Conjugation experiments were set up to assess whether ENR resistance was transferable to *E. coli* or *S. aureus* from Gram-negative and Gram-positive resistant strains, respectively. A previously described protocol [52] was used with modifications. The RIF-resistant strains derived from *E. coli* ATCC 25,922 and *S. aureus* ATCC 13,565 were used as recipients. Mid-logarithmic phase cultures (optical density at 625 nm between 0.4 and 0.5) of donor and recipient strains were mixed in a 1:10 ratio and placed onto a 0.22 μm membrane filter on LB plates and incubated at 27 °C overnight. Cells were collected in 1 mL of LB, and serial dilutions were spread on plates of LB agar supplemented with 100 μg/mL RIF and 10 μg/mL ENR. After spreading, plates were incubated at 27 °C for 48 h.

## Figures and Tables

**Table 1:** List of the enrofloxacin-resistant strains isolated from loggerhead sea turtles *Caretta caretta*.

Turtle	Isolate Code	Species	Resistance Profile ^1^	ENR MIC (μg/mL)	CST MIC (μg/mL) ^2^
A	FVBA1	*Escherichia coli*	AMP-ENR-STR-SXT-TET	32	2
	FVBA2	*Staphylococcus haemolyticus*	AMP-ENR-STR-SXT-TET	32	NA
C	FVBA15	*Escherichia coli*	ENR-STR-SXT	128	1
D	FVBA5	*Pseudomonas mendocina*	AMP-CHL-ENR-STR-SXT	128	2
E	FVBA6	*Pseudomonas mendocina*	AMP-CHL-ENR-STR-SXT-TET	128	2
F	FVBA8	*Citrobacter portucalensis*	AMP-CHL-ENR	16	2
G	FVBA10	*Escherichia coli*	AMP-CTX-FEP-ENR	128	2
	FVBA9	*Escherichia coli*	AMP-CTX-ENR-SXT-TET	128	2
H	FVBA11	*Escherichia coli*	AMP-CTX-ENR-STR	128	1
	FVBA12	*Escherichia coli*	AMP-CTX-ENR-STR-SXT-TET	256	1
I	FVBA16	*Vibrio fluvialis*	AMP-ENR-SXT-TET	64	1
J	FVBA13	*Pseudomonas mendocina*	AMP-CHL-ENR-SXT	128	2
M	FVBA19	*Morganella morganii*	AMP-CST-ENR-TET	128	256
N	FVBA42	*Enterococcus faecium*	AMP-CTX-ENR-FEP-GEN-IPM-STR	64	NA
	FVBA46	*Vagococcus fluvialis*	AMP-CTX-ENR-FEP-STR-SXT-TET	32	NA
O	FVBA40	*Vagococcus fluvialis*	AMK-CTX-ENR-FEP-STR-SXT-TET	32	NA
P	FVBA26	*Citrobacter freundii*	AMP-CHL-ENR-SXT-TET	32	2
	FVBA36	*Vagococcus fluvialis*	CTX-ENR-FEP-STR-SXT-TET	32	NA
	FVBA25	*Citrobacter freundii*	AMP-CHL-ENR-SXT-TET	32	2
Q	FVBA37	*Vagococcus fluvialis*	AMK-CHL-CTX-ENR-FEP-GEN-STR-SXT-TET	32	NA
	FVBA38	*Vagococcus fluvialis*	CHL-ENR-STR-SXT	32	NA
R	FVBA27	*Aeromonas caviae*	AMP-ENR-TET	32	2
	FVBA33	*Vagococcus fluvialis*	CHL-CTX-ENR-FEP-STR-SXT-TET	32	NA
	FVBA39	*Vagococcus fluvialis*	AMK-ENR-FEP-GEN-STR-SXT-TET	32	NA
S	FVBA29	*Klebsiella variicola* subsp. *variicola*	AMP-CTX-ENR-FEP-GEN-STR-SXT-TET	32	2
	FVBA32	*Vagococcus fluvialis*	AMP-CTX-ENR-FEP-GEN-STR-SXT-TET	32	NA
T	FVBA23	*Citrobacter freundii*	AMP-CHL-ENR-SXT-TET	32	2
	FVBA43	*Vagococcus fluvialis*	ENR-STR-SXT	32	NA
U	FVBA22	*Citrobacter freundii*	AMP-CHL-ENR-SXT-TET	32	2
	FVBA41	*Vagococcus fluvialis*	CTX-ENR-FEP-STR-SXT-TET	32	NA
V	FVBA44	*Vagococcus fluvialis*	ENR-FEP-STR-SXT	32	NA
	FVBA45	*Aeromonas veronii*	AMP-ENR	32	2
W	FVBA30	*Vagococcus fluvialis*	ENR-FEP-STR-SXT	32	NA
X	FVBA47	*Pseudomonas mendocina*	AMP-CHL-ENR-FEP-SXT-TET	64	2
Z	FVBA24	*Citrobacter freundii*	AMP-CHL-ENR-IPM-SXT-TET	32	2
	FVBA31	*Vagococcus fluvialis*	AMK-ENR-FEP-STR-SXT	32	NA

^1^ AMK: amikacin; AMP: ampicillin; CHL: chloramphenicol; CST: colistin; CTX: cefotaxime; ENR: enrofloxacin; FEP: cefepime; GEN: gentamicin; IPM: imipenem; STR: streptomycin; SXT: sulfamethoxazole/trimethoprim; TET: tetracycline. ^2^ NA: Not applicable to Gram-positive species.

## Data Availability

The sequence data are available in the NCBI GenBank database at the accession number PQ763953. The MALDI-TOF profiles of the identified strains will be made available by the authors on request. All the other data are included in the article.
